# What drives people to play pickleball? A mixed-methods study using SEM and fsQCA

**DOI:** 10.3389/fpsyg.2026.1740931

**Published:** 2026-02-10

**Authors:** Wenzhe Huang, Duxiang Xiao, Bojin Cheng

**Affiliations:** 1 School of Arts and Sports, Shantou Preschool Education College in Guangdong, Shantou, China; 2 School of Physical Education, Guangzhou Sport University, Guangzhou, China

**Keywords:** exercise motivation, theory of planned behavior, pickleball, fuzzy-set qualitative comparative analysis, structural equation modeling

## Abstract

**Introduction:**

This study aims to elucidate the pathways through which behavioral intention for pickleball participation is formed among the general public (i.e., individuals excluding professional or competitive athletes) and to analyze the decision-making mechanisms underlying participation.

**Methods:**

Grounded in the Theory of Planned Behavior (TPB), this research introduces exercise motivation as an antecedent variable. Based on a survey of 315 pickleball participants, it employs a mixed-methods approach combining Structural Equation Modeling (SEM) and fuzzy-set Qualitative Comparative Analysis (fsQCA) to empirically examine the influencing factors, pathways, and causal configurations of participation intention.

**Results:**

(1) Exercise motivation significantly and positively influences behavioral intention; (2) Attitude and perceived behavioral control fully mediate the relationship between exercise motivation and behavioral intention, whereas the mediating role of subjective norm is not significant; (3) The inclusion of exercise motivation effectively enhances the explanatory power of the extended TPB model in predicting behavioral intention; (4) fsQCA identifies two distinct configurations sufficient for triggering high behavioral intention, with a positive attitude serving as a core condition in both.

**Discussion:**

This study broadens the application boundaries of TPB in the context of sports participation and enriches the research on the psychological mechanisms of public participation in pickleball. Using the case of China, it provides theoretical support and practical guidance for promoting and popularizing pickleball in regions where the sport is in its early stages of development.

## 1 Introduction

Pickleball is an emerging paddle sport that combines elements of badminton, tennis, and table tennis. In recent years, it has gained rapid popularity in Western countries due to its low entry barrier, moderate physical intensity, cost-effectiveness, and strong social appeal (Jia et al., 2024). Having been recognized as the fastest-growing sport in the United States for three consecutive years, pickleball was included as a demonstration sport in the 2024 Paris Olympics (Zhang, 2024). In contrast, its development in China remains at a nascent stage. The establishment of the China Pickleball Working Committee in late 2023 reflects organized efforts to promote and standardize the sport (Huang, 2023). Nevertheless, a persistent challenge in promoting pickleball lies in translating initial casual interest into sustained participation, highlighting the need to investigate the psychological drivers of long-term engagement.

The Theory of Planned Behavior (TPB) has been widely used to explain and predict sports participation (Fang, 2011; Feng and Mao, 2014). However, a notable limitation of TPB is its insufficient attention to the internal drives that precede and energize the formation of its core constructs, namely attitude, subjective norm, and perceived behavioral control (Hagger et al., 2022). Furthermore, the predictive strength of these constructs, particularly subjective norm, is known to vary across behavioral contexts. This gap is especially pertinent in voluntary, leisure-oriented settings.

Pickleball provides an ideal context to examine these theoretical issues due to its low level of institutionalization. Unlike established racket sports (e.g., tennis) that operate within formal structures and ingrained social norms, pickleball is characterized by community-driven and leisure-oriented participation. Comparative research confirms this distinction, noting that pickleball’s simplified rules and minimal entry barriers foster a more informal and accessible participation ecology compared to tennis (Ma and Zhou, 2025). This distinctive, low-pressure environment likely attenuates the influence of subjective norms, thereby elevating the relative importance of individual exercise motivation and personal attitudes in shaping participation decisions. Therefore, to more comprehensively explain behavioral intention in such a context, it is essential to integrate exercise motivation as a key antecedent within an extended TPB framework, as it may initiate and shape the subsequent cognitive evaluations captured by TPB (Feng et al., 2015).

Furthermore, prior research has predominantly employed symmetric methods such as Structural Equation Modeling (SEM), which assumes linear and additive relationships. Such approaches may overlook the possibility that multiple concurrent configurations of motivational and cognitive conditions can lead to the same behavioral outcome. This limitation can be addressed through configurational techniques such as fuzzy-set Qualitative Comparative Analysis (fsQCA). The integrated SEM–fsQCA approach has been widely applied and validated across multiple disciplines, including sports participation (Huang, 2025; She et al., 2025), library and information science (Chen et al., 2025; Shen et al., 2025), and public health research (Feng et al., 2024; Liu and Yu, 2022), providing an established analytical framework for identifying multiple conjunctural causations and equifinal pathways. Accordingly, this study offers a dual-method contribution. First, it explicitly models exercise motivation as a distal antecedent within TPB to clarify how this internal drive activates subsequent cognitive evaluations. Second, it applies fsQCA to identify causal configurations associated with high behavioral intention, thereby revealing equifinal pathways that may not be captured by SEM alone.

From a practical perspective, understanding participation behavior is especially important for promoting pickleball in developing countries where the sport is still emerging. In contexts such as China, where pickleball has been incorporated into national fitness policies, research should prioritize uncovering the drivers of active recreational participation rather than competition-oriented involvement or spectatorship. A clear understanding of the psychological drivers underlying behavioral intention, particularly the role of exercise motivation, can thus provide a theoretical rationale to support the promotion and popularization of pickleball as an emerging sport.

## 2 Literature review

### 2.1 Theory of planned behavior and pickleball participation

The Theory of Planned Behavior (TPB), developed by Fishbein and Ajzen as an extension of the Theory of Reasoned Action, is one of the most extensively applied theoretical models for predicting exercise behavior (Fang, 2011; Feng and Mao, 2014). Its application in sports participation research primarily follows two trajectories. The first trajectory focuses on building and validating predictive or interventional models for general physical activity within specific populations, such as adolescents (Fang and Sun, 2010; Hagger et al., 2007). The second trajectory employs TPB to explain and predict participation in specific sports or leisure activities, including pickleball tournament participation (Wang et al., 2023), ice-snow tourism (Cao and Ge, 2024), and sports lottery purchases (Liu et al., 2014).

Theory of planned behavior posits that behavioral intention, the most proximal predictor of actual behavior, is shaped by three core antecedents: attitude (an individual’s positive or negative evaluation of performing the behavior), subjective norm (perceived social pressure from significant others), and perceived behavioral control (the perceived ease or difficulty of performing the behavior). While the predictive validity of intention is well-established, the relative influence of these antecedents can vary across behavioral contexts. For instance, research on ice-snow sports found subjective norm to be the strongest predictor for adolescents (Liang, 2022), whereas studies on lottery purchase identified attitude as the dominant factor (Liu et al., 2014). As a form of sports participation and leisure activity, the formation of behavioral intention among pickleball participants may share similarities with these findings. Nevertheless, the relative explanatory power of each antecedent within the context of pickleball requires further investigation. Based on the TPB framework, this study proposes the following Hypothesis:

*H1*: Attitude positively influences behavioral intention.

*H2*: Subjective norm positively influences behavioral intention.

*H3*: Perceived behavioral control positively influences behavioral intention.

### 2.2 Theory of planned behavior and exercise motivation

Despite its widespread application, the Theory of Planned Behavior (TPB) has well-documented limitations. Empirical evidence suggests that a significant portion of variance in behavioral intention remains unexplained by its three core antecedents alone (Armitage and Conner, 2001). Furthermore, the framework does not explicitly address the motivational origins of these antecedents, thereby limiting its explanatory depth regarding why individuals form specific behavioral intentions (Hagger and Chatzisarantis, 2009). To address this, scholars have attempted to extend the model by integrating complementary theoretical perspectives such as Self-Determination Theory. Within this integrative effort, the construct of motivation has garnered considerable attention due to its central role in initiating, guiding, and sustaining behavior (Feng and Mao, 2014; Wang et al., 2025). Meta-analytic studies (Hagger and Chatzisarantis, 2009) and empirical research in domains including innovation adoption (Zheng and Li, 2022) have demonstrated that motivation not only significantly influences behavioral intention but also shapes the three TPB antecedents.

Exercise motivation, defined as the psychological drive stimulating participation in physical activity (Chen et al., 2013), is a fundamental factor that may precede and shape the cognitive evaluations within TPB. Individuals with strong exercise motivation are more likely to develop a positive attitude toward a specific activity like pickleball, perceive greater social support or normative expectations, and feel more confident in their ability to participate. This view is supported by studies in related domains, such as those on sports event volunteers and esports users, where exercise motivation significantly predicted attitude (Ma and Wang, 2024; Zhang et al., 2023). Consequently, exercise motivation is posited not only to directly enhance participation intention but also to exert indirect influence through the TPB antecedents. Therefore, this study proposes the following Hypothesis:

*H4*: Exercise motivation positively influences attitude.

*H5*: Exercise motivation positively influences subjective norm.

*H6*: Exercise motivation positively influences perceived behavioral control.

*H7*: Exercise motivation positively influences behavioral intention.

Furthermore, prior research integrating motivational constructs with TPB has established an extended theoretical framework wherein motivation influences behavioral intention through the mediation of cognitive and perceptual evaluations, namely the TPB constructs (Feng et al., 2015; Ge et al., 2020). As a form of physical activity, pickleball participation is conceptually situated within this established integrative framework. However, given the distinctive characteristics of pickleball, which include its emerging status, strong recreational and social appeal, and the typically community-driven rather than obligatory nature of participation, the specific mediating pathways through which exercise motivation influences the intention to play pickleball require further empirical examination, with the role of subjective norm being of particular interest. Accordingly, this study posits and aims to test the following mediation Hypothesis:

*H8*: Attitude mediates the relationship between exercise motivation and behavioral intention.

*H9*: Subjective norm mediates the relationship between exercise motivation and behavioral intention.

*H10*: Perceived behavioral control mediates the relationship between exercise motivation and behavioral intention.

**Figure 1:**
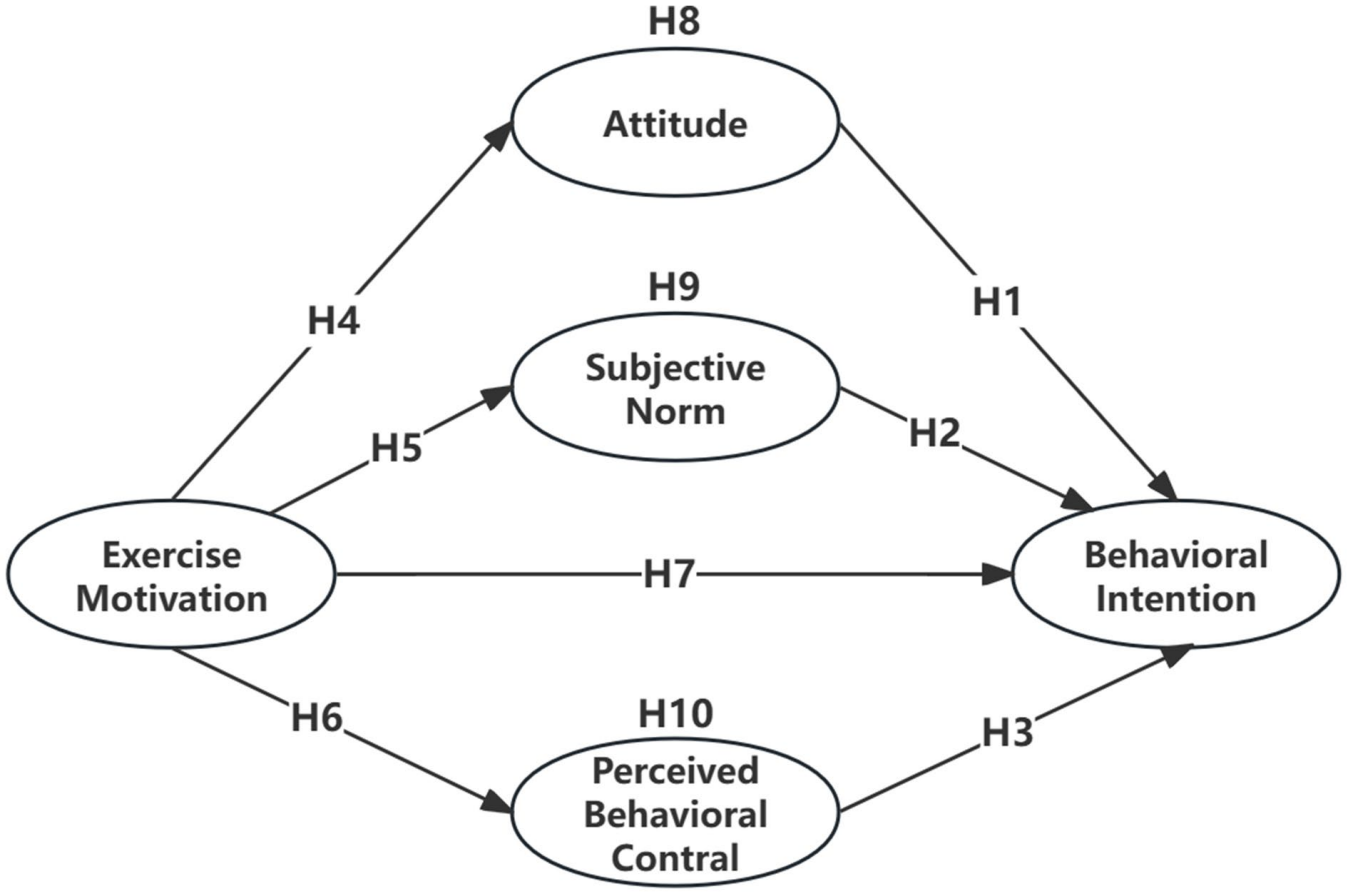
Research model of factors influencing pickleball participation behavioral intention.

Accordingly, based on the above discussions, this study proposes a research model to investigate the factors influencing behavioral intentions of recreational pickleball participants, as illustrated in Figure 1.

## 3 Research methods

### 3.1 Participants and data collection

This study investigates the behavioral intentions of recreational pickleball participants and their influencing factors. Data were collected between January and March 2025 using a mixed-mode survey strategy combining online and offline questionnaires. Offline data were gathered through convenience sampling at four established pickleball venues in Guangzhou and Shenzhen, China, where on-site players were randomly invited to participate. This approach facilitated direct access to active participants, thereby enhancing the relevance of responses—a recognized advantage of convenience sampling in exploratory research contexts (Etikan et al., 2016). Online data collection employed snowball sampling, with the questionnaire distributed through social media and dedicated pickleball enthusiast groups to extend reach to a broader and more varied network of participants, including those engaged in informal and socially oriented play—a method particularly effective for accessing otherwise hard-to-reach populations (Naderifar et al., 2017). While these non-probability sampling approaches may limit the generalizability of the findings, they are well-suited for exploratory investigations focusing on emerging and previously under-researched groups.

A total of 360 questionnaires were distributed. After excluding responses with excessively short completion times or clear patterned answers, 315 valid questionnaires were retained, yielding a valid response rate of 87.5%. The sample consisted of 53.3% male and 46.7% female participants. Regarding age distribution: 2.5% were under 18 years old, 36.3% were 18–25 years old, 18.3% were 26–33 years old, 10.5% were 34–41 years old, 11.7% were 42–49 years old, and 20.6% were 50 years or older.

### 3.2 Measurement instruments

The development of the questionnaire followed a systematic process. First, based on the research objectives and characteristics of pickleball, established scales from domestic and international literature were reviewed to draft initial items. Second, in-depth interviews were conducted with both casual and experienced pickleball participants, and the items were refined based on their feedback. Finally, six experts in sports science and psychology evaluated the content validity of the questionnaire, all of whom rated it as good or higher.

The final questionnaire comprised two sections: the first collected demographic information, and the second measured the core constructs, including exercise motivation, attitude, subjective norm, perceived behavioral control, and behavioral intention.

#### 3.2.1 Exercise motivation scale

The Motivation for Physical Activity Measure–Revised (MPAM-R), developed by Ryan et al. (1997) and adapted into Chinese by Chen et al. (2013), was used to assess exercise motivation. This scale consists of 15 items across five dimensions: health motivation (e.g., “I want to maintain a healthy body”), appearance motivation (e.g., “I want to maintain or improve my physique”), enjoyment motivation (e.g., “I want to participate in highly enjoyable activities”), competence motivation (e.g., “I want to improve my existing sports skills”), and social motivation (e.g., “I want to meet new friends”). All items were rated on a 5-point Likert scale ranging from 1 (strongly disagree) to 5 (strongly agree), with intermediate values of 2, 3, and 4. Higher total scores indicate stronger exercise motivation.

#### 3.2.2 TPB scales

As noted by Ajzen (2006), standardized TPB scales are not universally applicable due to the high specificity of behavioral contexts; instead, scales should be tailored to the behavior and setting under investigation. Accordingly, this study adapted established TPB scales from both domestic and international literature to fit the context of pickleball participation as an emerging sport. Attitude was measured using three items (e.g., “Participating in pickleball is enjoyable”) based on Xu and Qiao (2020) and Hagger et al. (2002). Subjective norm was assessed with three items (e.g., “Most people who are important to me would approve of my participation in pickleball”) derived from Ajzen (1991), Guo et al. (2013), and Deng (2012). Perceived behavioral control was evaluated using four items (e.g., “I am capable of mastering the necessary techniques and strategies in pickleball”) adapted from Xia and Shi (2025) and Dong et al. (2024). Behavioral intention was measured with three items (e.g., “I plan to continue participating in pickleball in the future”) based on Guo et al. (2013) and Hagger et al. (2002). All items were scored on a 5-point Likert scale from 1 (strongly disagree) to 5 (strongly agree), with intermediate ratings of 2, 3, and 4.

### 3.3 Data analysis

Data analysis was performed using SPSS 27.0 for reliability analysis, descriptive statistics, correlation analysis, and hypothesis testing. Amos 28.0 was employed for common method bias (CMB) testing and confirmatory factor analysis (CFA), with the bootstrap method used to examine the proposed mediation model. fsQCA 3.0 software was applied for qualitative comparative analysis of the condition configurations influencing the dependent variable.

#### 3.3.1 Common method bias test

As this study relied solely on self-reported questionnaires, CMB was a potential concern. To mitigate its impact and ensure the robustness of the results, both procedural and statistical remedies were implemented, following established methodological recommendations.

In terms of procedural controls, several measures suggested by Podsakoff et al. (2012) were adopted. First, respondents were informed that there were no right or wrong answers and that all items were clearly phrased to avoid ambiguity. The order of items was systematically arranged, and the specific constructs being measured were not disclosed to reduce hypothesis-guessing. Second, participants were assured that their responses would remain anonymous, used solely for academic purposes, and that their teachers would not have access to individual responses. These steps were taken to minimize social desirability bias and encourage honest answers.

For statistical control, the CFA-based approach recommended by Tang and Wen (2020) was applied. A single-factor model incorporating all measurement items was first constructed and compared against the theoretical multi-factor CFA model. The fit indices for the single-factor model were unsatisfactory: χ^2^/df = 4.853, GFI = 0.677, NFI = 0.719, IFI = 0.763, TLI = 0.743, CFI = 0.762, RMSEA = 0.111. As shown in Table 1, with 224 degrees of freedom and a 95% confidence interval, the lower bound of Δχ^2^ was 259.914. The observed Δχ^2^ in this study was 1248.564, substantially exceeding this critical value. These results indicate that common method bias did not pose a significant threat in this study.

**Table 1:** Changes in chi-square and degrees of freedom for CFA model comparisons.

**Model**	***χ*** ^***2***^	***df***	**△*χ*** ^***2***^	**△*df***	***p***
Single-factor	1698.454	350	1248.564	224	0.05
Multi-factor	449.976	126			

#### 3.3.2 Reliability, validity, and confirmatory factor analysis

Given the satisfactory model fit indices (χ^2^/df = 2.724, RMSEA = 0.074, CFI = 0.904, TLI = 0.885, IFI = 0.905), reliability and validity assessments were conducted. First, internal consistency was evaluated using Cronbach’s *α*. As shown in Table 2, all constructs exhibited values between 0.754 and 0.891, exceeding the recommended threshold of 0.7, indicating good reliability. Next, construct validity was assessed using composite reliability (CR) and average variance extracted (AVE). Values of CR greater than 0.7 and AVE greater than 0.5 are considered indicative of satisfactory convergent validity. The results summarized in Table 2 demonstrate that all CR values ranged from 0.826 to 0.945, and all AVE values fell between 0.616 and 0.852, confirming adequate composite reliability and convergent validity for all measured constructs.

**Table 2:** Results of reliability and validity analysis.

Variable	Item	Factor loading	CR	AVE	Cronbach’s α
Exercise motivation	hm1	0.922	0.945	0.852	0.891
	hm2	0.908			
	hm3	0.939			
	am1	0.887	0.826	0.616	0.767
	am2	0.727			
	am3	0.729			
	em1	0.925	0.888	0.729	0.791
	em2	0.902			
	em3	0.719			
	cm1	0.920	0.888	0.726	0.813
	cm2	0.867			
	cm3	0.762			
	sm1	0.859	0.918	0.788	0.872
	sm2	0.886			
	sm3	0.917			
Attitude	at1	0.897	0.906	0.764	0.819
	at2	0.900			
	at3	0.823			
Subjective norm	sn1	0.840	0.84	0.636	0.754
	sn2	0.770			
	sn3	0.781			
Perceived behavioral control	pbc1	0.824	0.88	0.648	0.795
	pbc2	0.770			
	pbc3	0.884			
	pbc4	0.735			
Behavioral intention	bi1	0.913	0.92	0.794	0.84
	bi2	0.858			
	bi3	0.901			

## 4 Results

### 4.1 Correlation analysis

Table 3 presents the descriptive statistics and correlations among the latent variables. The mean values of exercise motivation, attitude, subjective norm, perceived behavioral control, and behavioral intention ranged from 4.158 to 4.402, with standard deviations below 0.7, indicating low variability and good model fit. Correlation analysis revealed significant positive relationships among all latent variables. As shown in Table 3, the square roots of the AVE for all constructs were greater than their correlations with other latent variables, demonstrating adequate discriminant validity.

**Table 3:** Correlation analysis of variables.

Correlation	M	SD	EM	AT	SN	PBC	BI
EM	4.190	0.612	0.861				
AT	4.402	0.668	0.730**	0.874			
SN	4.158	0.679	0.636**	0.643**	0.797		
PBC	4.162	0.677	0.686**	0.688**	0.722**	0.805	
BI	4.301	0.675	0.702**	0.746**	0.644**	0.704**	0.891

**p < 0. 01; ***p < 0.001; Values on the diagonal represent the square roots of the AVE. c: EM = exercise motivation, AT = attitude, SN = subjective norm, PBC = perceived behavioral control, BI = Behavioral intention.

### 4.2 Structural equation modeling analysis

#### 4.2.1 Path coefficient testing of the hypothesized model

First, the overall model fit of the hypothesized model was analyzed using AMOS software. The obtained model fit indices were: χ^2^/df = 3.226, RMSEA = 0.084, CFI = 0.918, TLI = 0.901, IFI = 0.918, NFI = 0.886. All these indices reached the acceptable or good level according to the model fit criteria, indicating a good model fit for the study. The results of the SEM showed that the explanatory power of the TPB model for behavioral intention was *R*^2^ = 0.68. After adding the exercise motivation variable, the explanatory power for behavioral intention became *R*^2^ = 0.82. Compared with the TPB model, the change rate in the explanatory power of the expanded TPB model was Δ*R*^2^ = 0.14.

Mediation analysis was conducted using Hayes’s PROCESS macro (Model 4) to test the study’s Hypothesis. The results demonstrated that attitude (*β* = 0.369, *p* < 0.001), subjective norm (*β* = 0.110, *p* = 0.033), perceived behavioral control (*β* = 0.231, *p* < 0.001), and exercise motivation (*β* = 0.227, *p* < 0.001) all exerted significant direct effects on behavioral intention, thereby providing support for Hypothesis H1, H2, H3, and H7. Furthermore, exercise motivation showed significant positive effects on attitude (*β* = 0.797, *p* < 0.001), subjective norm (*β* = 0.709, *p* < 0.001), and perceived behavioral control (*β* = 0.760, *p* < 0.001), confirming Hypothesis H4, H5, and H6.

#### 4.2.2 Mediation effect analysis

The bootstrap method, recognized as an effective approach for testing multiple mediation effects (Lau and Cheung, 2012), was employed to examine the mediating roles of attitude, subjective norm, and perceived behavioral control. Using 5,000 bootstrap samples, indirect effects were considered statistically significant if their 95% bias-corrected confidence intervals did not include zero. As summarized in Table 4: (1) The indirect effect through attitude was 0.294, with a 95% CI [0.186, 0.401], indicating significant mediation and supporting H8. (2) The indirect effect through subjective norm was 0.077, with a 95% CI [−0.003, 0.167], indicating non-significant mediation. Thus, H9 was not supported. (3) The indirect effect through perceived behavioral control was 0.176, with a 95% CI [0.073, 0.288], confirming significant mediation and supporting H10. Notably, the mediating effect of attitude was stronger than that of perceived behavioral control.

**Table 4:** Results of the mediation effect test.

**Type of Effect**	***β***	**SE**	**LLCI**	**ULCI**	**Proportion Mediated**
Total Effect	0.774	0.044	0.687	0.862	
Direct Effect	0.227	0.059	0.111	0.344	29.33%
Total Indirect Effect	0.547	0.071	0.409	0.688	70.67%
EM → AT→BI	0.294	0.055	0.186	0.401	37.98%
EM → SN → BI	0.077	0.043	−0.003	0.167	
EM → PBC → BI	0.176	0.054	0.073	0.288	22.74%

Abbreviations see note to Table 3.

### 4.3 Fuzzy-set qualitative comparative analysis

While SEM focuses on the net effects of linear relationships, it does not account for interactions among variables or their combined effects. In contrast, fsQCA is particularly suited for analyzing outcomes arising from multiple concurrent causes (Du and Jia, 2017). Therefore, to complement the SEM findings and enhance the depth and robustness of the results, this study employed fsQCA to examine the configurations of antecedent conditions influencing behavioral intentions toward pickleball participation. Based on theoretical and empirical foundations, four antecedent conditions were selected: exercise motivation, attitude, subjective norm, and perceived behavioral control.

#### 4.3.1 Data calibration

All variables involved in the analysis were calibrated using the direct method. Following Ragin’s (2008) recommendation, the 5th, 50th, and 95th percentiles of each antecedent condition were used as thresholds for full non-membership, the crossover point, and full membership, respectively, to enhance the interpretability of the results (Ragin, 2008).

#### 4.3.2 Analysis of necessary conditions

Necessity analysis was conducted for each individual antecedent condition. A condition was considered necessary if its consistency score exceeded 0.9 (Lv et al., 2024). As shown in Table 5, none of the conditions reached this threshold, indicating that no single antecedent is sufficient to explain behavioral intention. Consequently, a configurational analysis combining multiple conditions was warranted.

**Table 5:** Results of necessary condition analysis.

**Condition**	**Consistency**	**Coverage**
EM	0.810	0.854
~EM	0.449	0.572
AT	0.858	0.836
~AT	0.339	0.570
SN	0.787	0.859
~SN	0.452	0.570
PBC	0.813	0.859
~PBC	0.435	0.553

Abbreviations see note to Table 3.

#### 4.3.3 Analysis of sufficient configurations

Considering the relatively large sample size, the analysis set a consistency threshold of 0.8, a PRI consistency threshold of 0.7, and a case frequency threshold of 3 to avoid configurations with limited empirical relevance. The analysis yielded complex, intermediate, and parsimonious solutions. Based on the nested relationships between the intermediate and parsimonious solutions, core and peripheral conditions were identified, with the resulting configurations presented in Table 6.

**Table 6:** Results of configurational analysis.

**Condition**	**Causal Recipes for High BI**	
	**Path 1**	**Path 2**
EM	●	
AT	●	●
SN		●
PBC		•
Raw Coverage	0.733	0.649
Unique Coverage	0.138	0.054
Solution Consistency	0.901	0.920
Solution Coverage	0.787	
Solution Consistency	0.887	

● Indicates the presence of a core condition; •indicates the presence of a peripheral condition; “Blank” indicates that the condition’s presence or absence is irrelevant to the configuration (the condition can be either present or absent); Abbreviations see note to Table 3.

In line with established practice (Lv et al., 2024), configurations with consistency above 0.8 and coverage above 0.5 were deemed acceptable. As shown in Table 6, two configurations were identified as sufficient for generating high behavioral intention. The individual consistency scores were 0.901 and 0.920, respectively, and the overall solution consistency was 0.887, indicating that the two configurations are sufficient conditions for high behavioral intention. The overall solution coverage was 0.787, suggesting that these configurations explain approximately 78.7% of the cases with high behavioral intention. These results demonstrate the strong explanatory power of the model.

#### 4.3.4 Robustness checks

To assess the robustness of the fsQCA results and minimize potential randomness, robustness checks were performed by adjusting key analytical thresholds, as recommended in prior studies (Zhang and Du, 2019). Specifically, the consistency threshold was increased from 0.8 to 0.9, and the case frequency threshold was raised from 3 to 5. The resulting truth table and configurations were then compared with the original results. The findings showed that the core conditions and configurations leading to high behavioral intention remained consistent. Moreover, changes in consistency and coverage were minimal and did not lead to substantively different interpretations. These results confirm the robustness of the identified configurations for explaining high behavioral intention in recreational pickleball participation.

## 5 Discussion

### 5.1 The significant positive impact of exercise motivation on behavioral intention: the core internal drive for pickleball participation

Exercise motivation is a pivotal determinant of behavioral intention in pickleball participation. This finding corroborates existing evidence on the positive link between motivation and exercise intention (Ge et al., 2020). More specifically, the results indicate that within the emerging, low-structure context of pickleball, participation intention is strongly driven by intrinsic exercise motives. Unlike traditional racket sports underpinned by established organizational structures and systematic coaching pathways, pickleball engagement appears to be primarily fueled by autonomous motivations, such as the pursuit of health, enjoyment, social connection, or skill mastery. This outcome aligns with the Self-Determination Theory principle that autonomous motivation more effectively sustains behavioral engagement. It further implies that promoting such a sport requires strategies aimed at activating and nurturing these internal drivers.

### 5.2 The full mediating roles of attitude and perceived behavioral control: the psychological and practical bridges to pickleball participation

Attitude and perceived behavioral control play crucial and fully mediating roles in the mechanism influencing pickleball participation intention, consistent with prior findings (Zhang et al., 2023). This pattern of full mediation indicates that, in this context, exercise motivation must first translate into positive cognitive-affective evaluations and a sense of capability before shaping a concrete intention to participate. Specifically, attitude, which captures an individual’s cognitive and affective evaluation of participating in pickleball, serves as the critical translator. A positive attitude leads individuals to perceive the activity as enjoyable and beneficial, thereby strengthening their intention to engage. Simultaneously, perceived behavioral control, reflecting the individual’s assessment of the resources and abilities needed to perform the behavior, acts as a vital enabler. The accessible nature of pickleball, characterized by simple equipment and easy-to-learn rules, effectively reduces technical anxiety and lowers the initial barrier to entry. This accessibility enhances participants’ sense of self-efficacy and controllability. Consequently, promotion strategies must recognize that merely stimulating motivation is insufficient; it is equally essential to cultivate positive subjective experiences and to design participation environments that reinforce individuals’ confidence in their ability to succeed.

### 5.3 The non-significant mediating role of subjective norm: the autonomous nature of pickleball participation

The results indicate that subjective norm did not significantly predict pickleball participation intention, and its mediating role between exercise motivation and behavioral intention was non-significant. This finding differs from some conclusions in prior physical activity research. For instance, a meta-analysis in health contexts found a significant, albeit small-effect, mediating role of self-determined motivation on intention via subjective norm (Hagger and Chatzisarantis, 2009). However, it aligns with observations from other studies applying TPB in contexts emphasizing personal volition, such as research on Chinese university students’ extracurricular physical exercise, where subjective norm similarly showed no significant effect (Xia and Shi, 2025). The attenuated role of subjective norms in this study can be attributed to an interplay of activity-specific, cultural, and demographic factors.

First, regarding activity type, pickleball as an emerging leisure sport is characterized by self-organized community engagement and participation driven primarily by personal interest. Unlike institutionalized or health-normative physical activities, its appeal lies in enjoyment and social interaction, where individual autonomy tends to outweigh perceived social pressure (Wang et al., 2023). Second, in terms of cultural and demographic context, our sample was drawn from metropolitan residents in Guangzhou and Shenzhen—economically developed and culturally open cities in China. This socio-cultural milieu, often associated with higher individualism and greater exposure to novel lifestyle choices, may further diminish the salience of traditional social influences on individual behavioral decisions.

Consequently, the findings suggest that the predictive strength of subjective norm within the TPB framework is not universal but is contingent upon behavioral and contextual factors. This does not refute the TPB but underscores the need for its context-sensitive refinement, acknowledging that the relative importance of its core constructs may vary across behavioral domains and cultural settings. It is therefore pertinent that this study employs fsQCA to further investigate whether and how subjective norm operates conditionally within specific configurations of personal and social factors.

### 5.4 The introduction of exercise motivation as an antecedent variable: effectively enhancing the TPB model’s explanatory power

The SEM results of this study show that the traditional TPB model explained 68% of the variance in behavioral intention toward pickleball participation among participants, reaffirming its universal value in the domain of individual behavioral decision-making. After introducing exercise motivation as an antecedent variable, the expanded TPB model explained 82% of the variance in behavioral intention, representing a 14% increase over the traditional TPB. This outcome suggests that while the traditional TPB model can explain part of the mechanism behind intention formation, the inclusion of exercise motivation renders the prediction of behavioral intention more precise and comprehensive, particularly in the context of emerging sports characterized by distinct individualistic features. Theoretically, integrating exercise motivation upgrades the traditional TPB framework to a dynamic system that outlines a pathway from being motivation-driven, through psychological cognition, to intention formation. This systemic perspective aligns well with the strong autonomy and diverse needs inherent in pickleball participation.

### 5.5 Integration of SEM and fsQCA findings: toward a nuanced theoretical understanding

While the preceding SEM analysis identified the average strength and mediation pathways within the extended TPB model, the fsQCA results provide a crucial complementary perspective by uncovering multiple, distinct pathways to high behavioral intention.

Specifically, fsQCA identified two sufficient causal recipes. The first path centers on the core combination of high exercise motivation and a positive attitude, representing an internally-driven route. The second path hinges on a positive attitude together with strong subjective norms, supported by perceived behavioral control, illustrating a socially-embedded route.

The theoretical significance of these configurational findings is twofold. First, they robustly establish a positive attitude as a necessary, core condition across all solutions, confirming its paramount role. Second, and more importantly, they reveal the conditional nature of variable influences. While SEM indicated a non-significant average mediating effect for subjective norm, fsQCA demonstrates that it can operate as a core causal element within the specific social-contextual configuration (Path 2). This suggests that the influence of social norms is not universally weak but is activated decisively only when combined with a positive personal attitude and adequate perceived control. Similarly, the presence of different core conditions across paths shows that high intention can be driven either by strong internal motivation or by a supportive social environment, a complexity that linear models typically do not capture.

Thus, the two methods offer a dialectical insight. SEM effectively maps the central tendencies and primary mechanisms. fsQCA, in turn, explains the heterogeneity underlying these averages by showing how the same outcome can be achieved through different logical combinations of conditions. This integration resolves the apparent paradox of subjective norm’s role, moving from a conclusion of “no general effect” to a more precise understanding of its contextual importance. Together, they advance a more complete and contingent theoretical framework for understanding participation in emerging sports.

## 6 Implications and limitations

### 6.1 Theoretical contributions

This study advances the Theory of Planned Behavior (TPB) and its application to emerging, voluntary recreational sports through three key theoretical contributions. First, it extends the TPB by establishing exercise motivation as a critical distal antecedent. This specification addresses a core theoretical limitation by elucidating the motivational origins of the model’s cognitive and social antecedents, thereby significantly enhancing its explanatory power for behaviors driven primarily by personal volition rather than external mandates. Second, it refines the understanding of social influence within TPB by clarifying the conditional role of subjective norm. The findings advance the theoretical perspective from viewing subjective norm as a universally predictive factor to recognizing it as a contextually activated core condition, thereby delineating important boundary conditions for its operation. Third, it moves beyond the conventional linear paradigm of TPB research. While prior studies have predominantly focused on linear relationships among variables, our integrated SEM–fsQCA approach explicitly accounts for configurational effects. This provides a more nuanced understanding of participation decisions and establishes a methodological pathway for capturing causal complexity in behavioral research.

### 6.2 Practical implications

Based on the core findings of this study, which identify exercise motivation, positive attitude, and perceived behavioral control as key psychological drivers of behavioral intention toward pickleball, promotion strategies should focus on systematically stimulating intrinsic motivation, shaping positive initial experiences, and lowering barriers to entry. Specifically, promotional content and activity design should directly address participants’ diverse internal motives by highlighting pickleball’s benefits for health and appearance motivation, such as its role in improving cardiorespiratory function, coordination, and body composition; emphasizing its dynamic and enjoyable gameplay to satisfy enjoyment motivation; leveraging its doubles-dominated format and community atmosphere to meet social motivation; and offering clear skill progression pathways to fulfill competence motivation. In practice, priority should be given to creating low-cost, easily accessible “first-experience” scenarios, such as utilizing existing public spaces through minimal infrastructural modifications. It is essential to ensure that beginners receive guided support to achieve enjoyable and successful initial encounters, thereby rapidly fostering a positive attitude. Concurrently, structured onboarding guidance, positive skill transfer from related sports, and the establishment of peer support networks should be provided to reduce technical anxiety and perceived difficulty, effectively enhancing participants’ sense of control and self-efficacy. Supporting measures, including training community instructors, developing accessible event formats, and integrating information resources, can provide the necessary foundation for these core psychological strategies. Collectively, these measures establish a cohesive framework that supports the progression from motivational impetus to sustained behavioral engagement.

### 6.3 Limitations and future research

However, this study has several limitations. First, the primary use of cross-sectional survey data limits the ability to definitively establish causal relationships among variables. Future research could employ longitudinal tracking or experimental methods to further validate the findings. Second, the sample size was relatively limited, and data collection was confined to specific regions within China. Variations in policy support, economic levels, cultural context, and geographical climate across different regions might lead to differentiated mechanisms influencing pickleball participation intention. For instance, from a policy dimension, varying levels of government support for emerging sports and investment in public sports facilities directly affect the role of facility accessibility as an external condition within perceived behavioral control. Economically, disparities in disposable income and sports consumption capacity between developed and developing countries might result in regional differences in the strength of exercise motivation’s influence. Therefore, future studies could adopt stratified regional sampling and multi-group comparison analysis to contrast the strength of macro-regional characteristic influences and the generalizability of the findings.

## 7 Conclusion

1. Exercise motivation demonstrates a significant positive impact on the intention to engage in pickleball.
2. Attitude and perceived behavioral control fully mediate the relationship between exercise motivation and pickleball participation intention. In contrast, the mediating effect of subjective norm is found to be statistically non-significant.
3. The extended Theory of Planned Behavior model, which incorporates exercise motivation as an antecedent variable, provides a robust predictive framework for explaining behavioral intention toward pickleball participation. Furthermore, this extended model offers a substantial improvement in explanatory power for behavioral intention compared to the traditional TPB model.
4. The fsQCA results reveal two distinct causal configurations sufficient for triggering a high level of participation intention. The first path is characterized by the combination of high exercise motivation and a positive attitude as core conditions. The second path features a positive attitude and strong subjective norms as its core conditions, with perceived behavioral control acting as a peripheral condition in this configuration.

## Data Availability

The raw data supporting the conclusions of this article will be made available by the authors, without undue reservation.
